# Avian and Mammalian Facilitative Glucose Transporters

**DOI:** 10.3390/microarrays6020007

**Published:** 2017-04-05

**Authors:** Mary Shannon Byers, Christianna Howard, Xiaofei Wang

**Affiliations:** 1 Department of Biological Sciences, 3500 John A. Merritt Blvd., Tennessee State University, Nashville, TN 37209, USA; maryshannonbyers@yahoo.com; 2 Department of Biology, Langston University, Langston, OK 73050, USA; cahoward@langston.edu

**Keywords:** glucose transporter, GLUTs, chicken, avian, mammal, phylogenetic analysis

## Abstract

The GLUT members belong to a family of glucose transporter proteins that facilitate glucose transport across the cell membrane. The mammalian GLUT family consists of thirteen members (GLUTs 1–12 and H^+^-myo-inositol transporter (HMIT)). Humans have a recently duplicated GLUT member, GLUT14. Avians express the majority of GLUT members. The arrangement of multiple GLUTs across all somatic tissues signifies the important role of glucose across all organisms. Defects in glucose transport have been linked to metabolic disorders, insulin resistance and diabetes. Despite the essential importance of these transporters, our knowledge regarding GLUT members in avians is fragmented. It is clear that there are no chicken orthologs of mammalian GLUT4 and GLUT7. Our examination of GLUT members in the chicken revealed that some chicken GLUT members do not have corresponding orthologs in mammals. We review the information regarding GLUT orthologs and their function and expression in mammals and birds, with emphasis on chickens and humans.

## 1. Introduction

The GLUTs are a family of glucose transporter proteins that transport glucose bidirectionally across cell membranes by way of facilitative diffusion [1,2]. They are members of the solute carrier family 2A (*slc2a*). GLUTs are composed of 12 membrane-spanning helices with regions in the extracellular matrix and cytoplasm and contain several functionally conserved motifs [3,4]. In humans, the GLUT family consists of GLUTs 1–12 and H^+^-myo-inositol transporter (HMIT). HMIT is also known as GLUT13 [5]. Humans also have additional expression of GLUT14, which is a duplicon of GLUT3 [6]. Based on phylogenetic analyses, GLUTs are divided into three classes. Class I is made up of GLUTs 1–4, and GLUT14 in humans. Class II consists of GLUTs 5, 7, 9 and 11. Class III contains GLUTs 6, 8, 10, 12 and HMIT/GLUT13 [5]. It is widely believed that all members of the GLUT family originated from a common ancestor through duplication. During evolution, duplicated members acquired specialty such that they may either develop substrate specificity, or could be regulated in specialized ways that are advantageous to the species. In cells, multiple GLUTs are arranged in a tissue-specific manner, exhibiting different kinetic and regulatory properties [5,7]. All ectopically expressed GLUT members have demonstrated the ability to facilitate hexose transport [5], while some are specific to the transport of urate, myo-inositol or fructose. Fructose transport is especially important due to metabolic abnormalities acquired from high concentrations of fructose in the diet [8]. Defects in glucose and fructose transport are associated with insulin resistance, diabetes [9] and hyperfructosemia [10].

GLUT expression patterns are complex features. Much attention has been focused on characterizing mammalian GLUT members and elucidating their specific physiological roles. Several studies have also examined the role of GLUTs among avian species, which have provided a basis for understanding GLUT expression patterns in various tissues during different stages of development. However, due to the underlying complex nature of GLUTs, despite the progress made in GLUT research, our knowledge about avian GLUTs is fragmented. For example, the exact physiological role is still not clear for several GLUTs, and even the tissue specificity of GLUTs is not fully examined. In this article, we review what is known about facilitative GLUT family members and their function and expression in birds, with particular emphasis on chickens, and the mammalian (especially human’s) GLUTs as a starting point for a better understanding.

## 2. Glucose Transport

After the breakdown of dietary polysaccharides, glucose, fructose and galactose are taken up by enterocytes lining the microvilli of the small intestine. GLUT5 on the lumenal surface of the small intestine mediates fructose uptake. Sodium-dependent glucose cotransporters (members of the sodium-glucose cotransporter (SGLT) protein family) mediate the uptake of glucose and galactose. GLUT2 on the basolateral surface of enterocytes facilitates the release of hexoses into the circulatory system for reuptake by other cells [11]. When monosaccharide levels are high, GLUT2 may facilitate hexose uptake from the gut lumen [12]. In hepatocytes and other somatic cells, GLUT5 mediates fructose uptake from the circulatory system. Phosphorylation by tissue-specific kinases converts cytosolic glucose to glucose-6-phosphate (G6P). The negative charge on G6P prevents it from crossing the cell membrane. Glucokinase, which has a low affinity for glucose and is not inhibited by G6P, catalyzes this reaction in hepatocytes. When blood glucose concentrations are high, hepatocytes may accumulate G6P to buffer glucose concentrations. Glucose-6-phosphatase allows G6P from gluconeogenesis and glycogen breakdown to exit liver and kidney cells. Hexokinase isoforms, which have a high affinity for glucose and are feedback inhibited by G6P, catalyze the reaction of glucose to G6P in other body tissues. Those tissues can take up glucose during times when blood glucose concentrations are low. However, they are not able to accumulate high levels of G6P. The absence of glucose-6-phosphatase makes glucose uptake irreversible in those tissues [13,14].

## 3. GLUT Transporter Classes

### 3.1. Class I GLUTs

GLUTs 1–4, and GLUT14 in human, make up the Class I family of glucose transporters. In mammalian species, the *glut1/slc2a1* gene encodes the major GLUT protein of the blood–brain barrier [15]. The encoded protein is located primarily along the cell surface and in the cell membrane. GLUT1 may be responsible for constitutive or basal glucose uptake in cells and can transport a wide range of aldoses, including pentose and hexose [16,17]. On the cell surface, human GLUT1 may function as a receptor for T-cell leukemia virus I and II. Gene mutations associated with GLUT1 deficiency in humans have been linked to microcephaly and childhood epilepsy [18,19], hypoglycorrhachia [20,21], cryohydrocytosis with reduced stomatin [22], paroxysmal dystonic choreathetosis [23], episodic ataxia [22], hemiplegic migraines [24,25], spasticity and paroxysmal exertion-induced dyskinesia [26]. Overexpression of GLUT1 was shown to be an indicator for cancer [27] and to have an association with thymic carcinoma [28]. Suppression of GLUT1 by apigenin slowed overexpression of GLUT1 and had anticancer properties in mouse lung cancer cells [29]. Chicken GLUT1 shares 80% amino acid residues with humans [30]. Chicken GLUT1 has ubiquitous expression, with abundant expression in the hypothalamus, and has demonstrated response to insulin and dexamethasone [31]. According to the National Center for Biotechnology Information (NCBI) Gene Database [32], *glut1/slc2a1* orthologs are conserved in 124 organisms including human, chicken, chimpanzee, cow, mouse, rat, Rhesus monkey, zebrafish and *Eremothecium gossypii* (fungus).

In mammals, *glut2/slc2a2* encodes a glycoprotein. The encoded protein regulates bidirectional glucose transport across liver cells, pancreatic islet beta cells that store and release insulin, epithelial kidney cells and intestines. Similar to mammalian species, chickens have abundant GLUT2 expression in the liver [33], pancreatic beta cells, kidney and small intestine [34]. Due to its low affinity for glucose, GLUT2 may be a glucose sensor. *glut2/slc2a2* gene mutations in humans are associated with increased disease susceptibility, including noninsulin-dependent diabetes mellitus and Fanconi–Bickel syndrome. Mutations in *glut2/slc2a2* were also found to increase risk of cardiovascular disease in patients with type 2 diabetes [35,36]. Alternative gene splicing results in multiple transcript variants. Based on the NCBI Gene Database [32,37], *glut2/slc2a2* orthologs have been found in 168 organisms including human, chicken, dog, chimpanzee, cow, Rhesus monkey, rat, *Xenopus tropicalis* (western clawed frog), *Xenopus laevis* (African clawed frog) and zebrafish.

Mammalian GLUT3 facilitates the uptake of glucose, 2-deoxyglucose, galactose, mannose, xylose, fucose and other monosaccharides across the cell membrane. GLUT3 does not mediate fructose transport [36,38]. GLUT3 deficiency has been implicated in age of onset in Huntington’s disease [39]. Chicken GLUT3 is known to be a neuronal glucose transporter and shares 70% sequence similarity with that of humans [2]. The neuronal functions of GLUT1 and GLUT3 are similar across chickens and mammals [30,31]. In chickens, the upregulation of GLUT1 and GLUT3 is associated with the formation of tight junctions in the blood-retinal barrier [40]. Orthologs of *glut3/slc2a3* are preserved across 70 organisms so far, including chicken, dog, cow, chimpanzee, mouse, rat, Rhesus monkey, *X. tropicalis*, *X. laevis*, zebrafish, fruit fly, mosquito, *Caenorhabditis elegans* (non-parasitic roundworm), *Saccharomyces cerevisiae* (yeast), *Kluyveromyces lactis* (yeast), rice, *Magnaporthe oryzae* (rice blast fungus), *Neurosporra crassa* (red bread mold) and *Arabidopsis thaliana* (flowering plant), according to the NCBI Gene Database [32,37].

It is well known that GLUT4 is the major insulin sensitive glucose transporter in mammals. The mechanism by which insulin regulates GLUT4 activity has been well studied. Upon stimulation by insulin, intracellular GLUT4 translocates to the plasma membrane, where GLUT4 facilitates cellular glucose uptake. This constitutes the major portion of insulin-stimulated glucose uptake, especially in adipose tissue, skeletal muscle and cardiac muscle tissues. Humans and most mammals rely on normal protein expression of GLUT4 for blood glucose homeostasis [41]. *glut4* gene mutations in humans are associated with type 2 diabetes mellitus [42]. According to the NCBI Gene Database, *glut4/slc2a4* orthologs are found in 114 organisms including dog, cow, chimpanzee, mouse, rat and Rhesus monkey [32,37]. Chickens intrinsically lack *glut4* expression, and chickens are known to be naturally hyperglycemic with adipose tissue [2,43,44] and skeletal muscle tissue [45] that is poorly sensitive to insulin.

GLUT14, a duplicon of GLUT3, has been shown to have messenger RNA (mRNA) expression in the human testis [6] and, according to the NCBI Gene Database, may have a specific function related to spermatogenesis in males [46]. One study linked a polymorphism of *slc2a14* to having a possible role in the development of late-onset Alzheimer’s disease in a Han Chinese population [47]. High GLUT14 expression was also found to be associated with gastric adenocarcinoma [48]. According to the NCBI Gene Database, *slc2a14* orthologs are present in humans and Western gorillas [32,37]. In *Oryctolagus cuniculus* (rabbit), *slc2a14* is known as proteins GLUT3 and SLC2A14. In Rhesus monkey, the *LOC715795* gene is known as proteins SLC2A3 and SLC2A14. *slc2a3b* orthologs are also present in zebrafish. UniProt lists *slc2a1* as the gene that encodes the GLUT14 protein in *X. tropicalis*, inferred from database entries.

### 3.2. Class II GLUTs

Class II consists of GLUTs 5, 7, 9 and 11. GLUT5 is a fructose transporter protein with expression across many species [49]. According to the NCBI Gene and Protein databanks, human GLUT5 is thought to be a cytochalasin β-sensitive carrier with expression in human testis, spermatozoa, small intestine [49], adipose tissue and skeletal muscle [50]. More recent RNA-seq analyses found human GLUT5 expression in duodenum, bone marrow and kidney [51]. GLUT5 was found to have an association with malignant clear renal cell carcinoma [52]. According to the NCBI Gene database [32,37], orthologs of *glut5/slc2a5* are found so far in 123 organisms across chicken, dog, cow, chimpanzee, Rhesus monkey, mouse, rat and *X. tropicalis*. Chicken GLUT5 has been shown to have mRNA expression in the small intestine [53] and may be regulated by glucocorticoids [54].

GLUT7 has been identified as a high affinity transporter for glucose and fructose. GLUT7 does not transport galactose, 2-deoxyglucose or xylose [55]. Human GLUT7 has expression in the small intestine and colon, with lower expression levels in the testis and prostate [55]. Based on our searches of the NCBI Gene and Protein Database and UniProt Database [37,46,56], there are no data for GLUT7 in chickens or other avian species, suggesting that the avian lineage has lost *slc2a7* during evolution. Orthologs of *slc2a7* are conserved in 55 organisms across mouse, rat, chimpanzee and Rhesus monkey, according to the NCBI Gene Database.

GLUT9 is a known transporter of fructose and urate and can transport glucose at a low rate. Mammalian GLUT9 plays a regulatory role in the development and survival of cartilage chondrocytes and may have a role in urate reabsorption by proximal tubules [57,58]. One study linked gout to GLUT9 deficiency in a population of Japanese males [59]. It is assumed that chicken GLUT9 mediates uric acid uptake, although substrate specificity for this GLUT transporter has not yet been identified [33]. Liver mRNA expression of GLUT9 was shown to be greater in obese chickens, possibly due to having a larger glucose uptake capacity with greater demand and glucose load in high bodyweight chickens [33]. Based on the NCBI Gene Database [32,37], two transcript variants with distinct isoforms have been identified for *glut9/slc2a9*. Orthologs of *glut9/slc2a9* are present in 153 organisms including chicken, dog, cow, mouse, rat, chimpanzee, *X. tropicalis* and *X. laevis*.

According to the NCBI Gene Database, GLUT11 is also known as GLUT10. GLUT11 is a transporter of glucose and fructose, but does not transport galactose in humans. GLUT11 has roughly 42% amino acid sequence similarity to GLUT5 and 35% similarity to GLUT1 [60]. Alternative splicing results in multiple transcript variants, including GLUT11-A, GLUT11-B and GLUT11-C [61]. Mammalian GLUT11-A has expression in skeletal muscle, heart and kidney. Mammalian GLUT11-B is expressed in adipose tissue, kidney and placenta. Mammalian GLUT11-C has expression in skeletal muscle, heart, adipose tissue and pancreas [62]. Based on NCBI RefSeq, there is also evidence of a fourth GLUT11 isoform, known as GLUT11-D [46]. Human *glut11/slc2a11* orthologs are present in 111 organisms and conserved across chicken, dog, cow, chimpanzee, Rhesus monkey, zebrafish and *X. tropicalis*, based on the NCBI Gene Database [37]. Rats and mice lack the *glut11/slc2a11* gene [62].

### 3.3. Class III GLUTs

Class III contains GLUTs 6, 8, 10, 12 and HMIT/GLUT13. According to NCBI, *slc2a6* has alias GLUT6 and GLUT9 proteins in humans, mice and *X. tropicalis*. GLUT6 is a hexose transporter protein. Mammalian GLUT6 is highly expressed in the brain, spleen and leukocytes [63]. One study linked an upregulation of GLUT6 to endometrial cancer in women [64]. Based on the NCBI Gene Database [32,37], *GLUT6/SLC2A6* orthologs are present in 169 organisms including chicken, dog, cow, mouse, chimpanzee, Rhesus monkey, zebrafish, fruit fly, mosquito, *X. tropicalis* and *X. laevis*.

Based on sequence similarity, GLUT8 has been identified as an insulin-regulated glucose transporter. According to NCBI, GLUT8 binds cytochalasin β in a glucose-inhibitable manner. Mammalian GLUT8 may be dual-specific and is inhibitable by fructose. A recent study on the mouse atria suggests that GLUT8 has a role in glucose uptake in the mammalian heart, along with GLUT4 [65]. *glut8/slc2a8* orthologs are conserved across 171 organisms including chicken, dog, mouse, rat, cow, chimpanzee, Rhesus monkey, *X. tropicalis*, zebrafish, fruit fly, *A. thaliana* and rice, according to NCBI. Similar to mammals, chicken GLUT8 is a known insulin-responsive glucose transporter with ubiquitous expression in cells and higher mRNA concentrations in adipose tissue and kidney [1].

According to the NCBI Gene Database, GLUT10 plays a role in glucose homeostasis regulation. Human GLUT10 has highest mRNA expression in the liver and pancreas [66]. In humans, genetic mutations of *glut10/slc2a10* are associated with arterial tortuosity syndrome, a rare connective tissue disorder [67]. Based on NCBI, *glut10/slc2a10* orthologs are conserved across 166 organisms including chicken, dog, mouse, rat, chimpanzee, Rhesus monkey, cow, *X. tropicalis*, *X. laevis* and zebrafish [32,37].

According to the Gene Database at NCBI, the *slc2a12* encoded protein contains alias GLUT8 and GLUT12 in humans. GLUT12 can facilitate transport of a variety of hexoses [68]. Human GLUT12 is expressed in skeletal muscle, heart and prostate, with lower mRNA expression in the brain, placenta and kidneys [69]. A recent study implicated GLUT12 expression in the frontal cortex for its role in Alzheimer’s disease, a metabolic disease which impairs the brain’s ability to utilize glucose [70]. The GLUT12 level, as well as GLUT1 level, was shown to be elevated in hypertension and diabetic neuropathy in animal studies [71]. A recent study of GLUT12 in chicken skeletal and cardiac muscle suggests that GLUT12 may act as an insulin-sensitive transporter similar to GLUT4 in mammalian species [72]. Orthologs of *glut12/slc2a12* are conserved across 177 organisms including chicken, dog, mouse, rat, chimpanzee, Rhesus monkey, cow, *X. tropicalis*, *X. laevis*, zebrafish, *A. thaliana* and rice, based on the NCBI Gene Database [32,37].

Studies on *Xenopus* oocytes have helped identify GLUT13 as a proton (H^+^) myo-inositol cotransporter with specificity for the transport of myo-inositol, inositol triphosphate and related stereoisomers [73,74]. Mammalian HMIT/GLUT13 is predominantly expressed in glial cells and some neurons and may be responsible for myo-inositol brain metabolism regulation [73]. Intracellular function of HMIT may also be responsible for mood control [74]. Genetic alterations of HMIT may also be associated with non-small-cell lung cancer [75] and Parkinson’s disease [76]. According to the NCBI Gene Database [32,37], *glut13/slc2a13* orthologs are conserved across 151 organisms including chicken, dog, cow, chimpanzee, Rhesus monkey, mouse, rat, *X. tropicalis*, *X. laevis*, zebrafish, *C. elegans*, *S. cerevisiae*, *K. lactis*, *E. gossypii*, *Schizosaccharomyces pombe* (fission yeast), *A. thaliana* and rice.

## 4. GLUT Annotation

Automated computational analysis using genomic sequencing prediction method and contig reference sequencing has assisted in identifying *glut/slc2a* genes across different species. Through these methods, avian species *Struthio camelus australis* and *Anas platyrhynchos* have been shown to contain orthologs of *glut*s *1*, *2*, *3*, *5*, *6*, *8*, *9*, *10*, *11*, *12* and *hmit/glut13*. The turkey (*Meleagris gallopavo*) contains *glut*s *1*, *2*, *5*, *6*, *8*, *9*, *10*, *12* and *hmit/glut13*, but *glut3* and *glut11* have not yet been identified in turkey. From UniProt analysis, the duckbill mammal platypus (*Ornithorhynchus anatinus*) contains *glut* members *1*, *2*, *3*, *4*, *6*, *8*, *9*, *10*, *11*, *12* and *hmit/glut13*. However, these GLUT proteins remain uncharacterized in the species. From our analysis, *glut5* or *glut7* genes have not been identified in platypus.

It is not surprising that some GLUT members are annotated confusingly in public databases. Table 1 shows alias GLUT members GLUT6 and GLUT9, GLUT11 and GLUT10 and GLUT12 and GLUT8. The Gene Database at NCBI annotated *slc2a6* encoded protein as alias proteins GLUT6 and GLUT9 in human, mouse and *X. tropicalis*. Human *slc2a11* encoded protein was annotated as alias proteins GLUT10 and GLUT11. Clearly, *slc2a*6 and *slc2a*9 in human, mouse and *X. tropicalis* are discrete genes that encode discrete GLUT proteins; *slc2a10* and *slc2a11* in human are discrete genes which encode discrete GLUT proteins; and *slc2a*8 and *slc2a12* in human are also discrete genes which encode discrete GLUT proteins.

## 5. Evolutionary Relationships among GLUT Members

**Figure 1:**
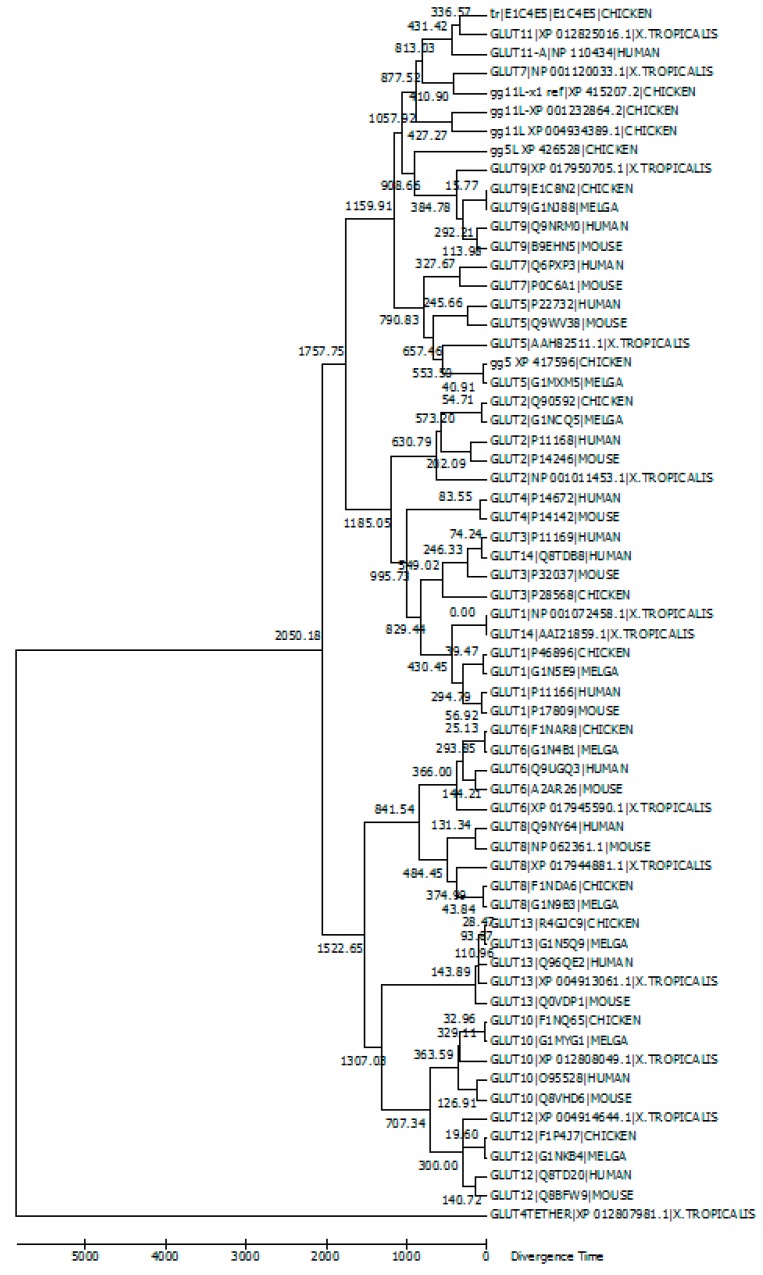
Evolutionary relationships of GLUTs. This evolutionary timetree was conducted in MEGA6 using the Neighbor–Joining bootstrap method. Each node represents a 95% confidence interval. Analysis included 63 GLUT amino acid sequences with a total of 426 positions in the final dataset. The timetree is drawn to scale with a relative number of substitutions per site. Based on this analysis and University of California Santa Cruz (UCSC) Genome Browser for *Gallus gallus*, accession number gg5L_X1_XP_426528.4 is a gene product that is discrete from the other GLUT5 members in chicken and other species and has more similarity to GLUT9 transporters. Key: *MELGA* is turkey (*Meleagris gallopavo*).

Figure 1 presents a timetree, which was constructed with Molecular Evolutionary Genetics Analysis Version 6.0 (MEGA6) software [77], for GLUT members from human, mouse, chicken, turkey (*Melga*) and *X. tropicalis*. The tree was calibrated with GLUT12, assuming the time of separation between mammals and birds was 300 million years. The human GLUT11-A was included in the phylogenetic analysis. This analysis was conducted using the Neighbor–Joining bootstrap method with 50 replicates. Topological branching point divergence times were calculated with maximum likelihood based on the Jones–Taylor–Thornton matrix-based method and are based on units of the number of amino acid substitutions per site.

GLUT amino acid sequences were downloaded from UniProt or NCBI and derived from evidence at the transcript level, protein level or homology. Dataset for the timetree contained 63 amino acid sequences with a total of 426 positions included in the final dataset. Positions with fewer than 95% site coverage were eliminated. Less than 5% alignment gaps, missing data and ambiguous bases were allowed at any position. The timetree is drawn to scale with the relative number of substitutions per site.

According to this timetree, Class III GLUTs separated from Class I and Class II approximately 2000 million years ago. Class I and Class II GLUTs separated about 1700 million years ago, around the time when multicellular life began. GLUTs 1 and 3 separated approximately 800 million years ago. Evidently, GLUT13/HMIT orthologs could not be resolved well with this tree construction method, which can be seen from the distance between mammals and birds being not reflecting the species tree. GLUT8 and GLUT10 orthologs had the least constraints among the GLUT family members.

Based on the phylogenetic analysis as well as the result of experiments conducted in our laboratory, we have recognized that accession number gg5L_X1_XP_426528.4, which is annotated as GLUT member 5-like isoform X1 in chickens, is a gene product that is separate from the true GLUT5 member of chicken and other species and has more similarity to the GLUT9. Several GLUT11-like members were also found in chickens, each of them being the product of a discrete gene (Figure 1).

## 6. Chicken GLUT Members

Characteristics of human and chicken GLUT members are summarized in Table 2. The first chicken genome draft has helped to identify genes for GLUTs 1, 2, 3, 5, 8 and 9 in chicken [78]. GLUT12 was also recently examined in chicken skeletal and cardiac muscle [72]. For chicken studies, GLUT1 has been analyzed across various chicken tissues [33] and embryonic myoblasts [31] and fibroblasts [79]. *glut2* was cloned by screening a chicken liver cDNA library [34] and examined for expression in various chicken tissues [1,57]. GLUT3 was examined across various chicken tissues [1,30,33]. GLUT5 was examined for its presence in enterocytes and its mRNA expression pattern [55,57]. GLUT8 has been tested across various chicken tissues [1,2]. GLUT9 was tested in chickens [33]. GLUTs 6, 9, 10, 11, 12 and HMIT/GLUT13 were derived from orthology from a 2004 large-scale analysis comparing evolutionary conserved regions between chicken and mammalian genomes [78], but little information is available regarding their expression patterns.

GLUT expression patterns vary across species, and glucose transport may be regulated by different factors in those species. One such example is GLUT4, which is required for normal cellular metabolism in mammalian species, but is lacking in avian species.

A study from 1994 showed GLUT2 to be predominantly expressed in the chicken liver, but absent in the chicken brain and heart. Similar to mammalian species, this early study revealed how multiple GLUTs coexisted in various tissues in chickens. A 2001 study examined chicken mRNA and protein expression during different stages of embryonic development. GLUT1, GLUT3 and GLUT4 were examined in several tissues during embryogenesis. In the chicken brain, GLUT1 mRNA levels were high throughout development, although GLUT1 protein expression was highest during early development. GLUT3 mRNA expression in the brain was highest during the last half of development, with high protein expression very early and very late in development. In skeletal muscle, mRNA and protein expression of GLUT1 and GLUT3 were high during early development, but decreased by mid-development. GLUT1 mRNA and protein expression were also highest during early development, then declined steadily throughout development. GLUT1 mRNA levels were high in the liver, but protein expression of GLUT1 was not detectable. This study determined that GLUT1 is developmentally regulated in the chicken brain, heart and skeletal muscle. GLUT3 in the brain increased throughout the stages of development, but was absent in skeletal muscle. This study also revealed that GLUT4 was absent in chicken heart and skeletal muscle [80].

Because GLUT4 homologs were found to be lacking in chickens, GLUT8, an insulin-responsive GLUT in mammalian blastocytes [81], was tested across various chicken tissues. In 2003, a research team used reverse transcription polymerase chain reaction (RT-PCR), DNA sequencing and Northern and Southern blot analysis to identify GLUT8 mRNA expression in different chicken tissues. GLUT8 mRNA expression was barely detectable in chicken adipose tissue, liver, heart and skeletal muscle. This study revealed that GLUT8 mRNA expression was different from mammalian species, in which GLUT8 is expressed across most insulin-responsive tissues [2].

In a 2005 follow up study by the same team of researchers, GLUT1, GLUT2, GLUT3 and GLUT8 were examined for the extent of mRNA expression across different chicken tissues, this time using real-time quantitative polymerase chain reaction (RT-qPCR) with SYBR Green I fluorescence. In this study, GLUT1 mRNA expression was detected across most of the tissues examined, with highest concentrations in adipose tissue and brain. GLUT2 mRNA expression was detectable only in the liver and kidneys. GLUT3 was found to be highly expressed in the brain, and GLUT8 mRNA was ubiquitously expressed across all chicken tissues with higher expression in adipose tissue and kidneys [1].

A 2014 study found that high bodyweight chickens are generally compulsive feeders with a different food intake and blood glucose threshold sensitivity to insulin [82]. In this study, GLUTs were expressed differently across insulin-induced hypoglycemic high versus low bodyweight chickens. Expression of GLUT1, GLUT2 and GLUT3 mRNA was higher in the hypothalamus and liver across high bodyweight groups. Hypoglycemia in chickens correlated with reduced GLUT expression in the liver [82]. 

Relative comparison methods were used for comparing GLUT mRNA expression across different genes. Our laboratory has developed an absolute quantification protocol to study a panel of different GLUTs across multiple chicken tissues. The absolute quantification method has allowed us to compare mRNA levels across different GLUTs (i.e., GLUT1 compared to GLUT2) and different tissues. By calculating absolute copy number values for absolute quantification, we were able to compare GLUT mRNA expression patterns across a tissue panel of different GLUTs. Results showed that the mRNA levels of different GLUTs have clear tissue specificity; many GLUTs are expressed in the same tissues.

With sequence analysis and RT-qPCR, we have also found new *glut* genes in chickens, including a member very similar to human *glut9* and several members similar to mammalian *glut11*. Analysis of these paralogs revealed their distinct expression pattern.

## 7. Prospects and Conclusions

As is the case for many protein families containing multiple paralogs in a species, the GLUT family of proteins exerts essential physiological functions that need be delicately regulated. Multiple proteins with overlapping molecular functions provide a cushion for genetic, physiological and environmental disturbance. At the same time, tissue specific distribution of these transporters allows the fine tuning of expression to the tissue specific condition. Studies on the details of this fine regulation have just started. Much remains to be investigated. It would not be surprising if some day findings indicate that a specific GLUT is a limiting factor to the development of a specific cell type. Physiological and genomic evidence indicate that the regulation of glucose homeostasis in avians is distinct from the mammalian counterpart. Further examination of GLUT members in birds will shed new light on the function of these family.

## Figures and Tables

**Table 1:** Alias glucose transporter (GLUT) members.

Gene	Alias	Accession Number	Species	Chromosome	Exons	A.A.	Start	End	Span
*slc2a6*	6, 9	NP_060055.2	Human	9	11	507	133,472,024	133,479,059	7036
*slc2a9*		NP_064425.2	Human	4	24	540	9,826,400	10,021,429	195,030
*slc2a6*	6, 9	AAI41169.1	Mouse	2	10	443	27,021,917	27,027,905	5989
*slc2a9*		AAI38214.1	Mouse	5	20	523	38,351,086	38,483,364	132,279
*slc2a6*	6, 9	XP_017945590.1	*X. tropicalis*	Unknown	10	504	95,667	104,372	8706
*slc2a9*		XP_017950705.1	*X. tropicalis*	1	15	527	195,610,477	195,628,747	18,271
*slc2a10*		NP_110404.1	Human	20	8	541	5,931,524	5,933,981	2458
*slc2a11*	10, 11	NP_110434.3	Human	15	14	503	8,026,649	8,027,108	460
*slc2a8*		NP_055395.2	Human	9	11	477	127,397,231	127,407,246	10,016
*slc2a12*	8, 12	EAW47994.1	Human	6	7	617	133,991,158	134,052,480	61,323

**Table 2:** Characteristics of human and chicken GLUT members.

Gene	Orthologs	Human	Chicken
*glut1/slc2a1*	Conserved in human, chicken, chimpanzee, cow, mouse, rat, Rhesus monkey, zebrafish, *Eremothecium gossypii*. 122 organisms contain orthologs with *slc2a1* [46].	Blood-brain barrier [15]. Receptor for T-cell leukemia virus I and II [46]. Microcephaly, childhood epilepsy [18,19] , hypoglycorrhachia [20,21] , cryohydrocytosis [22], choreathetosis [23], ataxia [22], migraines [24,25] , spasticity, dyskinesia [26], indicator for cancer [27], thymic carcinoma [28].	Hypothalamus, basal glucose uptake, ubiquitous [31,34].
*glut2/slc2a2*	Conserved in human, chicken, dog, chimpanzee, cow, Rhesus monkey, rat, frog and zebrafish. 168 organisms have orthologs of *slc2a2* [46].	Glycoprotein, bidirectional transport in liver, islet beta cells, intestine, kidney, glucose sensor, gene mutations associated with susceptibility to disease, noninsulin-dependent diabetes, Fanconi–Bickel syndrome. Alternative splicing results in multiple transcript variants of this gene [33,34].	Fructose, galactose, liver, pancreas, small intestine, kidneys [1,33,57], insulin dependent [33].
*glut3/slc2a3*	Conserved in dog, cow, frog, chimpanzee, Rhesus monkey, mouse, rat, chicken, zebrafish, fruit fly, mosquito, *Caenorhabditis elegans*, *Saccharomyces cerevisiae*, *Kluyveromyces lactis*, *Magnaporthe oryzae*, *Neurosporra crassa*, *Arabidopsis thaliana* and rice. 70 organisms have orthologs of *slc2a3* [46]	Mediates uptake of glucose, 2-deoxyglucose, galactose, mannose, xylose, fucose and other monosaccharides across the cell membrane. Gene mutation associated with Huntington’s disease [39,40] .	Neurons [1,30], insulin dependent [33].
*glut4/slc2a4*	Conserved in chimpanzee, Rhesus monkey, dog, cow, mouse and rat. 114 organisms have orthologs of *slc2a4* [46].	Insulin-regulated. Upon insulin stimulation, GLUT4 translocates to cell surface to transport glucose across the cell membrane. Gene mutations are associated with noninsulin-dependent diabetes mellitus [41].	Not exist in chickens [2,80].
*glut5/slc2a5*	Conserved in chicken, dog, mouse, rat, chimpanzee, Rhesus monkey, cow and frog. 123 organisms have orthologs of *slc2a5* [46].	Thought to be cytochalasin β-sensitive carrier, expression in small intestine [49], adipose tissue, skeletal muscle [50], duodenum, bone marrow, kidney [51], renal cell carcinoma [52].	Fructose, small intestine [55,57].
*glut6/slc2a6*	Conserved in chicken, dog, cow, chimpanzee, mouse, Rhesus monkey, zebrafish, fruit fly, mosquito and frog. 169 organisms have orthologs of *slc2a6* [46].	GLUT6/GLUT9 [46], hexose transport [63], endometrial cancer [64].	Uncharacterized protein [78].
*glut7/slc2a7*	Conserved in mouse, rat, chimpanzee and Rhesus monkey. Orthologs found in 55 organisms [46].	Glucose, fructose transport, expression in small intestine and colon, lower levels in testis and prostate [55].	Not found in chickens.
*glut8/slc2a8*	Conserved in chicken, dog, mouse, rat, chimpanzee, Rhesus monkey, cow, zebrafish, fruit fly, rice, *A. thaliana* and frog. Orthologs found in156 organisms [46].	Insulin-regulated, binds cytochalasin β in glucose-inhibitable manner, may be dual-specific, as it is inhibitable by fructose [69].	Ubiquitous, especially in adipose tissue, kidneys, insulin response [1,2].
*glut9/slc2a9*	Conserved in chicken, dog, cow, chimpanzee, mouse, rat and frog. Orthologs found in 153 organisms [46].	Fructose, urate transport, and glucose at a low rate, urate reabsorption by proximal tubules, regulatory role in development and survival of chondrocytes [59].	Liver [33].
*glut10/slc2a10*	Conserved in chicken, dog, mouse, rat, chimpanzee, Rhesus monkey, cow, frog and zebrafish. Orthologs found in 166 organisms [46].	Liver and pancreas [66], glucose regulation, gene mutations are associated with arterial tortuosity syndrome [46].	Uncharacterized [78].
*glut11/slc2a11*	Conserved in chicken, dog, cow, chimpanzee, frog, Rhesus monkey and zebrafish and frog. Orthologs found in 111 organisms [46].	Glucose, fructose. 11-A: skeletal muscle, heart, kidney. 11-B: adipose tissue, kidney, placenta. 11-C: skeletal muscle, heart, adipose tissue, pancreas [62], 11-D [46].	Uncharacterized [78].
*glut12/slc2a12*	Conserved in chicken, dog, mouse, rat, chimpanzee, Rhesus monkey, cow, frog, zebrafish, rice and *A. thaliana*. Orthologs found in 177 organisms [46].	GLUT8/GLUT12 [46], skeletal muscle, heart, prostate, lower levels in brain, placenta, kidneys [69], wide variety of hexoses [68], Alzheimer’s, hypertension, diabetic neuropathy [71].	Insulin-sensitive. May act as GLUT4 in skeletal and cardiac muscle [72].
*glut13/slc2a13*	Conserved in chicken, dog, cow, chimpanzee, rice, Rhesus monkey, mouse, rat, frog, zebrafish, *C. elegans*, *S. cerevisiae*, *K. lactis*, *E. gossypii*, *Schizosaccharomyces pombe* and *A. thaliana.* Orthologs found in 151 organisms [46].	Glial cells and neurons [73], myo-inositol and related stereoisomers [74], non-small-cell lung cancer [75], Parkinson’s [76].	Uncharacterized.
*glut14/slc2a14*	2 organisms have orthologs of human *slc2a14* [46].	Spermatogenesis [46], Alzheimer’s disease [47], gastric adenocarcinoma [48].	N/A
